# Rolipram, a Selective Phosphodiesterase 4 Inhibitor, Ameliorates Mechanical Hyperalgesia in a Rat Model of Chemotherapy-Induced Neuropathic Pain through Inhibition of Inflammatory Cytokines in the Dorsal Root Ganglion

**DOI:** 10.3389/fphar.2017.00885

**Published:** 2017-12-04

**Authors:** Hee Kee Kim, Seon-Hee Hwang, Elizabeth Oh, Salahadin Abdi

**Affiliations:** Department of Pain Medicine, Division of Anesthesiology and Critical Care, The University of Texas MD Anderson Cancer Center, Houston, TX, United States

**Keywords:** neuropathic pain, DRG, rolipram, PDE4, pain behavior, chemotherapy, paclitaxel

## Abstract

Chemotherapy-induced neuropathic pain is a significant side effect of chemotherapeutic agents and is the most common reason for stopping chemotherapy. The aim of the present study was to find the major site and mechanisms of action by which rolipram, a selective phosphodiesterase-4 inhibitor, alleviates paclitaxel-induced neuropathic pain. Chemotherapy-induced neuropathic pain was induced in adult male Sprague-Dawley rats by intraperitoneal injection of paclitaxel on four alternate days. Rolipram was administered systemically or locally into the lumbar spinal cord, L5 dorsal root ganglion, sciatic nerve, or skin nerve terminal. The mechanical threshold, the protein level of several inflammatory cytokines, and the cellular locations of phosphodiesterase-4 and interleukin-1β in the dorsal root ganglion were measured by using behavioral testing, Western blotting, and immunohistochemistry, respectively. The local administration (0.03-mg) of rolipram in the L5 dorsal root ganglion ameliorated paclitaxel-induced pain behavior more effectively than did local administration in the other sites. Paclitaxel significantly increased the expression of inflammatory cytokines including tumor necrosis factor-α (2.2 times) and interleukin-1β (2.7 times) in the lumbar dorsal root ganglion, and rolipram significantly decreased it. In addition, phosphodiesterase-4 and interleukin-1β were expressed in the dorsal root ganglion neurons and satellite cells and paclitaxel significantly increased the intensity of interleukin-1β (2 times) and rolipram significantly decreased it. These results suggest that the major site of action of rolipram on paclitaxel-induced neuropathic pain in rats was the dorsal root ganglion. Rolipram decreased the expression of inflammatory cytokines in the dorsal root ganglion. Thus, phosphodiesterase-4 inhibitors may ameliorate chemotherapy-induced neuropathic pain by decreasing expression of inflammatory cytokines in the dorsal root ganglion.

## Introduction

Many chemotherapy drugs, including taxanes (paclitaxel, docetaxel), platinum-based agents (cisplatin, carboplatin, oxaliplatin), vinca alkaloids (vincristine, vinblastine, vindesine, vinorelbine), bortezomib, thalidomide, lenalidomide, suramin, and epothilones, produce a syndrome called chemotherapy-induced neuropathic pain, whose symptoms include ongoing burning pain, tingling, and numbness in the glove and stocking areas of the hands and legs (Massey et al., 2014; Kim et al., 2015b). Additionally, chemotherapy-induced neuropathic pain usually begin after multiple doses of chemotherapeutic agents and persists as a long-lasting sequela after cancer treatment is completed (Massey et al., 2014). Neuropathic pain is a dose-limiting side effect and is difficult to treat with widely used analgesic drugs such as non-steroidal anti-inflammatory drugs, antidepressants, and anticonvulsants (Massey et al., 2014).

Rolipram is a selective phosphodiesterase (PDE)-4 inhibitor (Kim et al., 2015a). The phosphodiesterases are a group of enzymes that degrade the phosphodiester bond of secondary messengers such as cyclic adenosine monophosphate (cAMP) and cyclic guanosine monophosphate and then terminate their own action.(Houslay and Adams, 2003) PDE4, which is mainly found in nerve cells and immune cells, hydrolyzes only cAMP (Houslay and Adams, 2003). Therefore, by inhibiting PDE4, rolipram increases the amount of cAMP in nerve cells and immune cells. cAMP is a ubiquitous secondary messenger that controls many cellular processes (Raker et al., 2016). In particular, increased levels of cAMP inhibit proinflammatory processes such as chemotaxis, degradation, and phagocytosis (Ottonello et al., 1995; Rossi et al., 1998; Pryzwansky and Madden, 2003; Pearse et al., 2004). In detail, increased cAMP inhibits migration of monocytes, adhesion molecule expression on leukocytes, and chemokine-induced chemotaxis (Harvath et al., 1991; Derian et al., 1995; Rossi et al., 1998; Aronoff et al., 2005). Thus, rolipram inhibits inflammation in both nerve cells and immune cells through increasing cAMP levels.

Previously, we reported that systemic injection and systemic infusion of rolipram ameliorated chemotherapy-induced neuropathic pain in rats (Kim et al., 2015a). However, the major anatomical sites and mechanisms of action of rolipram in chemotherapy-induced neuropathic pain have not yet been reported. Therefore, the aims of the present study were to determine (1) the major site or sites at which rolipram acts to ameliorate paclitaxel-induced neuropathic pain, (2) the mechanisms of action of rolipram on paclitaxel-induced neuropathic pain in rats, and (3) a better understanding of how PDE4 inhibition works to alleviate neuropathic pain will help to identify potential drug targets for chemotherapy-induced neuropathic pain.

## Materials and Methods

### Experimental Animals

All experimental protocols were approved by the Institutional Animal Care and Use Committee of The University of Texas MD Anderson Cancer Center (Houston, TX, United States) and were carried out in accordance with the National Institutes of Health’s Guide for the Care and Use of Laboratory Animals. We used adult male Sprague-Dawley rats (200–350 g; Harlan, United States) in this study. The rats were housed in groups of two or three in plastic cages with soft bedding and free access to food and water under a normal 12/12-h light-dark cycle, a temperature of 22 ± 2°C, and 40–55% humidity. All animals were acclimated in their cages for 1 week before the experiments.

### Paclitaxel-Induced Neuropathic Pain Model

To induce neuropathic pain in the rats, paclitaxel (GenDEPOT, United States) was dissolved in 4% dimethyl sulfoxide (DMSO) and 4% Tween 80 in sterile saline (2 mg/ml) just prior to injection. Paclitaxel (2 mg/kg/ml) was injected intraperitoneally on four alternate days (days 0, 2, 4, and 6; cumulative dose of 8 mg/kg) to induce painful peripheral neuropathy (Kim et al., 2010, 2016a). In a vehicle group of rats, vehicle (4% DMSO and 4% Tween 80 in saline, 1 ml/kg) was injected intraperitoneally on four alternate days. Mechanical hyperalgesia was measured as described below before the first paclitaxel injection and at various time points after injection.

### Behavioral Tests for Mechanical Hyperalgesia

All behavioral tests were conducted by the same blinded experimenter. To measure mechanical hyperalgesia, a calibrated set of von Frey filaments (Bioseb, United States/Canada) was used to measure foot withdrawal thresholds in response to mechanical stimuli. Briefly, the animals were placed in a plastic chamber on top of a mesh screen and the mechanical threshold of the hind paw was determined by the up–down method using von Frey filaments (0.45 – 14.45 g). A von Frey filament was applied to the most sensitive areas (the center of the paw or the base of the third or fourth toes) of the plantar surface of the left hind paw for 3–4 s (Chaplan et al., 1994). An abrupt withdrawal of the foot during stimulation or immediately after stimulus removal was considered to be a positive response. Withdrawal thresholds were determined using the up–down method (Dixon, 1980). The 50% threshold value was calculated from the pattern using the formula: 50% threshold = 10^(^*^X^*^+^*^kd^*^)^/10^4^, where *X* is the value of the final von Frey filament used in log units, *k* is the tabular value for the pattern of positive/negative responses, and *d* (0.22) is the mean difference between stimuli in log units. The investigator who conducted the behavioral tests did not know which animal received rolipram and which did not until the end of the study.

### Sedation Test

To determine whether local injection of rolipram induced sedation, the rats’ posture and righting reflexes were evaluated immediately after all behavioral tests. Posture was rated on a 0-to-4 scale where 0 indicated normal posture and 4 indicated flaccid atonia. Righting reflexes were rated on a 0-to-4 scale where 0 indicated struggle and 4 indicated no movement (Devor and Zalkind, 2001; Kim et al., 2004, 2016a).

### Catheter Implantation in the Left L5 Dorsal Root Ganglion

Catheters were implanted in the left L5 dorsal root ganglion (DRG) of the rats according to the Lyu method, with slight modification (Lyu et al., 2000). The rats were anesthetized using isoflurane (4% for induction, 3% for maintenance) in oxygen, and the hair was clipped from their backs. A midline incision was made at the L4–L6 spinal level, and the left L5 spinal nerve tracking through the intervertebral foramen was identified after separation of the left paraspinal muscles from the vertebrae. The left L4 vertebral foramen was cleaned by careful removal of connective tissues, and a small hole was made with a curved micro-pin on the top in the foramen. A 5-mm length of polyethylene tubing (PE-10, total 7 cm) was inserted into the small hole made by the micro-pin and placed near the L5 DRG; the tubing was secured to the muscles at multiple sites and fed subcutaneously to the mid-thoracic level in order to expose the tip at the dorsal midline position. The tip of the tubing was sealed with a needle blocker. The PE-10 tubing was covered with PE-60 tubing for protection, and the incision was closed. The rats were returned to their cages after they had recovered fully from the anesthesia. One week after catheterization, a test compound solution was injected. A 27-gauge needle attached to a 20-μl Hamilton syringe was inserted into the implanted tubing, and a 10-μl volume of test solution was injected slowly for about 10 s. The tubing was then flushed with 0.1 ml of saline from a Hamilton syringe. Behavioral tests were conducted before and at the following time points after injection: 0.5, 1, 1.5, 2, 3, 4, 5, and 6 h. After the experiment, the position of the catheter tip was confirmed by injecting 1% trypan blue into the catheter.

### Identification of Major Sites of Action of Rolipram

Rolipram was administered locally to various sites including the skin nerve terminal, sciatic nerve, L5 DRG, or spinal cord on day 20 after the first injection of paclitaxel, when paclitaxel-induced neuropathic pain behavior was fully developed. Twelve rats were divided into two groups (control and rolipram) for each site.

#### Nerve Terminal in Skin

The rats received a single injection of 0.03 mg rolipram (Sigma Chemical Company, United States) or of vehicle (0.6% DMSO in olive oil; 50 μl/injection) into a nerve terminal in the plantar surface of the left hind paw (**Table 1**). Behavioral tests were conducted before rolipram injection (baseline) and repeated at 0.5, 1, 1.5, 2, and 3 h after injection.

**Table 1:** Local administration of rolipram.

Site	Rolipram dose	*N*	Vehicle
L5 DRG	0.01 mg/10 μl	6	3% DMSO in
	0.03 mg/10 μl	6	olive oil
Sciatic nerve	0.01 mg/10 μl	6	3% DMSO in
	0.03 mg/10 μl	6	olive oil
Spinal cord by direct intrathecal injection	0.01 mg/50 μl	6	0.6% DMSO in


	0.03 mg/50 μl	6	olive oil
Nerve terminal in skin	0.01 mg/50 μl	6	0.6% DMSO in
	0.03 mg/50 μl	6	olive oil

#### Sciatic Nerve

The rats were placed under light anesthesia using isoflurane (3% for induction and 1.5% for maintenance). The rats received a single local injection of rolipram (0.03 mg) or of vehicle (3% DMSO in olive oil; 10 μl/injection) 1 mm proximal to the trifurcation of the sciatic nerve of the left hind paw via a 27-gauge needle with a Hamilton syringe (**Table 1**). Behavioral tests were conducted before rolipram injection (baseline) and repeated at 0.5, 1, 1.5, 2, and 3 h after injection.

#### L5 DRG

Each rat received a single local injection of rolipram (0.01, 0.03 mg) or of vehicle (3% DMSO in olive oil; 10 μl/injection) into the left L5 DRG through the implanted PE-10 tubing (**Table 1**). Behavioral tests were conducted before rolipram injection (baseline) and repeated at 0.5, 1, 1.5, 2, and 3 h after injection.

#### Spinal Cord by Direct Intrathecal Injection

Each rat received a single local injection of rolipram (0.03 mg) or of vehicle (0.6% DMSO in olive oil; 50 μl/injection) into the lumbar spinal cord via direct lumbar puncture (**Table 1**). The rats were placed under light isoflurane anesthesia (3% for induction and 1.5% for maintenance). A direct lumbar puncture was made by inserting a 27-gauge needle connected to a Hamilton syringe between the L5 and L6 vertebrae. When the needle insulted the matter of the spinal cord, the tail showed abrupt movement. Behavioral tests were conducted before injection (baseline) and repeated at 0.5, 1, 1.5, 2, and 3 h after injection.

#### Local Administration of Dibutyryl-cAMP (db-cAMP) in the L5 DRG

To find analgesic effects of c-AMP, db-cAMP, a cAMP analog, was locally administered in the left L5 DRG. On day 20 after paclitaxel administration, 12 rats were divided into two groups (control and db-cAMP). Each rat received a single local injection of db-cAMP (0.05 mg) or of vehicle (saline; 10 μl/injection) into the left L5 DRG through the implanted PE-10 tubing. Behavioral tests were conducted before db-cAMP injection (baseline) and repeated at 1, 2, 3, 4, 5, and 6 h after injection.

### Western Blot Analysis

To examine the levels of inflammatory cytokines in the DRG of rats, paclitaxel or vehicle was intraperitoneally injected on days 0, 2, 4, and 6 as described above. The L1–L6 DRGs were removed on day 20 after the first injection of paclitaxel or vehicle. For Western blotting, the lumbar DRGs were removed 1 h after the intraperitoneal injection of rolipram on day 20 because rolipram showed significant analgesic effects at 1 h in a previous report (Kim et al., 2015a). The rats were anesthetized deeply with 4% isoflurane and perfused with cold saline. The L1–L6 DRGs were removed and frozen immediately in liquid nitrogen (Kim et al., 2016a). The DRGs were homogenized in RIPA cell lysis buffer with a protease inhibitor (GenDEPOT, United States), and the homogenates were centrifuged at 14,000 rpm at 4°C. The supernatants were then loaded on 10% sodium dodecyl sulfate-polyacrylamide gels and transferred to polyvinylidene fluoride membranes. The blots were incubated with a primary antibody against interleukin (IL)-1β (1:1000; 17 KDa; Santa Cruz Biotechnology, United States), tumor necrosis factor alpha (TNF-α; 1:1000; 26 KDa; Abcam, United States), phosphorylated nuclear factor kappa B (NF-κB) (p-NFκB; 1:1000; 65 KDa; Cell Signaling Technology, United States), or GAPDH (1:1000; 37 KDa; Santa Cruz Biotechnology) overnight at 4°C. The blots were then incubated with anti-rabbit horseradish peroxidase-conjugated secondary antibody (1:5000; GenDEPOT) or anti-goat horseradish peroxidase-conjugated secondary antibody (1:5000; GenDEPOT) at room temperature for 1 h. The immunoblots were analyzed with a chemiluminescence detection system (GenDEPOT, United States). The blots were scanned using SPOT Advanced imaging software (version 5.0, A division of Diagnostic Instruments, Inc, United States) and Adobe Photoshop 8.0 (Adobe Inc., United States). For equalizing protein loading, GAPDH expression was used as a control. The band densities were quantified using Image J software (National Institutes of Health, United States). A region of the band was taken, and then the background was subtracted. The expression of a protein was quantified as the ratio of the expression level of that protein to the expression level of GAPDH in the same lane. Relative expression values were calculated by dividing the average expression level of a protein in the paclitaxel- or rolipram-treated group by the average expression level of the same protein in the vehicle-treated group.

### Immunohistochemical Analyses

To determine the localization of PDE4 and IL-1β in DRGs, the L5 DRGs were removed on day 20 after the first paclitaxel or vehicle injection. The L5 DRGs were removed 1 h after the intraperitoneal injection of rolipram (3 mg/kg) on day 20 for immunohistochemical experiments (Kim et al., 2015a).

For extraction of tissue for immunohistochemical analyses, the rats were deeply anesthetized with 4% isoflurane and transcardially perfused with cold saline followed by cold 4% paraformaldehyde (Kim et al., 2016b). The left and right L5 DRGs were removed, post-fixed, cryoprotected in 30% sucrose, cryosectioned to a thickness of 9 μm, and mounted on slides. The sections were incubated with combinations of the primary antibodies overnight at 4°C and then incubated with secondary antibodies conjugated with either Alexa Fluor 568 (red) or Alexa Fluor 488 (green) for 2 h at room temperature. The primary antibodies and concentrations used were the neuronal marker anti-NeuN (monoclonal anti-mouse, 1:50; GenDEPOT), anti-PDE4 (polyclonal anti-rabbit, 1:50; Santa Cruz Biotechnology), the satellite cell marker anti-glial fibrillary acidic protein (GFAP; polyclonal anti-mouse, 1:50; Santa Cruz Biotechnology), and anti-IL-1β (polyclonal anti-rabbit, 1:50; Santa Cruz Biotechnology). In addition, ProLong Diamond antifade mountant (Thermo Fisher Scientific, United States) was applied to the sections with or without the nuclear and chromosome marker 4′,6-diamidino-2-phenylindole (DAPI; Thermo Fisher Scientific, United States) for 1 day at room temperature. The sections were coverslipped and stored at -20°C until imaging.

The immunostained DRG sections were viewed using a CELENA^®^ S digital cell imaging system (Logos Biosystems, United States). For analysis of PDE4 and IL-1β co-localization with NeuN, GFAP, and DAPI, DRG sections from three rats were double-stained.

For IL-1β quantification, 3 DRG sections were selected per rat and 4 fields of view per section were selected by an experimenter under blind condition. Images were obtained using CELENA^®^ S digital microscope with X20 objective. For each section, IL-1β intensity was analyzed using Image J software.

### Statistical Analysis

Data were summarized as means with standard errors of the means for the behavioral tests and as means with standard deviations for Western blotting. To compare the results of behavioral tests, we used one-way ANOVA with one repeated factor (time) followed by Dunnett’s multiple comparison test or two-way ANOVA with one repeated factor (time) followed by Sidak’s multiple comparison test. To compare results of Western blotting and Immunohistochemistry, we used the Mann-Whitney U test. In all analyses, *P* < 0.05 was considered statistically significant. The study design used parallel groups and investigator blinding. The data were analyzed using GraphPad Prism 6 (GraphPad Software, United States).

## Results

### Sedation

All rats had scores of 0 on measures of posture and righting reflexes after local injections of rolipram, indicating that local injection of rolipram did not produce sedation. These data suggest that any increase in the mechanical threshold observed in rolipram-treated rats was indeed the result of their analgesic effect and not sedation.

### Major Site of Action of Rolipram in Paclitaxel-Induced Neuropathic Pain

**FIGURE 1:**
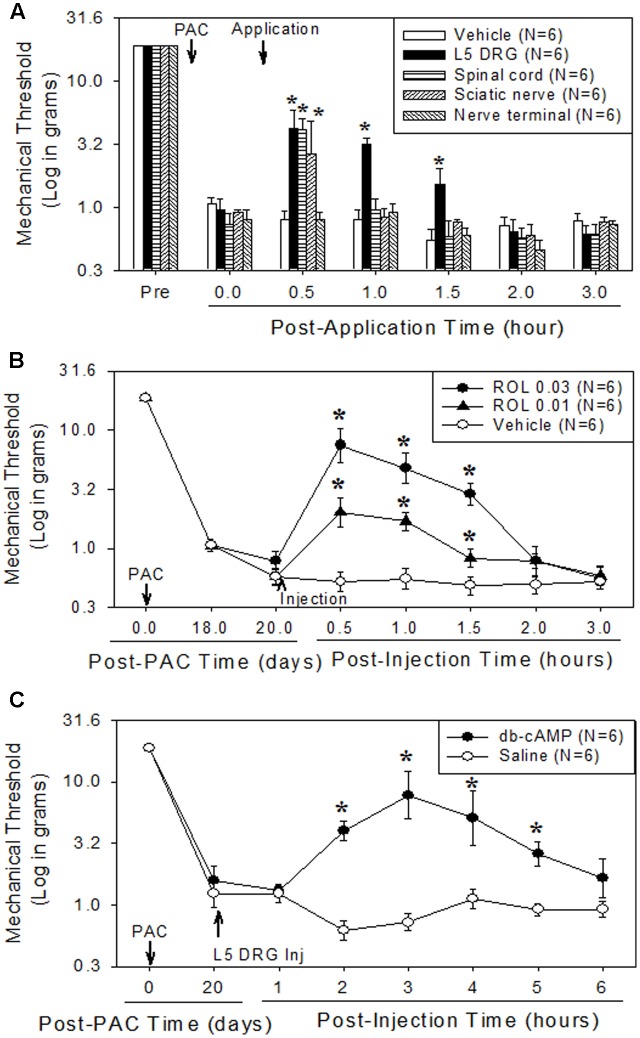
Comparison of the analgesic effect of local administration of rolipram (ROL) to the L5 DRG, spinal cord, sciatic nerve, and skin nerve terminal on established paclitaxel-induced neuropathic pain in rats. Paclitaxel (PAC, 2 mg/kg) was injected intraperitoneally in rats on four alternate days (days 0, 2, 4, and 6). Subsequently, the mechanical pain thresholds were significantly reduced. **(A)** [Analgesic effect of rolipram administered locally at four sites.] After the pain behavior was fully developed (20 days), 0.03 mg of rolipram was locally administered at four different sites. Local administration of rolipram at the L5 DRG significantly increased the mechanical pain threshold at 0.5, 1, and 1.5 h after administration. Vehicle was locally administered in the L5 DRG. Asterisks (^∗^) indicate significant differences (*P* < 0.05) from baseline as determined using a one-way ANOVA with one repeated factor followed by Dunnett’s multiple comparison test. **(B)** [Effect of local administration of rolipram in the L5 DRG] On the 20th post-paclitaxel injection day, the rats were divided into three groups (Vehicle, ROL 0.01, and ROL 0.03). Rats were administered vehicle (3% DMSO in olive oil, 10 μl), 0.01 mg of rolipram, or 0.03 mg of rolipram (indicated by the upward arrowhead), respectively. Administration of 0.03 mg of rolipram significantly increased the mechanical threshold at 0.5, 1, and 1.5 h. Asterisks (^∗^) indicate significant differences (*P* < 0.05) from vehicle group as determined using a two-way ANOVA with one repeated factor (time) followed by Sidak’s multiple comparison test. **(C)** [Effect of local administration of db-cAMP in the L5 DRG] On the 20th post-paclitaxel injection day, the rats were divided into two groups (Saline, db-cAMP). Rats in each group were administered saline or 0.05 mg of dibutyryl cAMP (db-cAMP, indicated by the upward arrowhead), respectively, in the L5 DRG. Administration of 0.05 mg of db-cAMP significantly increased the mechanical threshold at 2, 3, 4, and 5 h. Data are expressed as mean ± SEM. Asterisks (^∗^) indicate significant differences (*P* < 0.05) from saline group as determined using a two-way ANOVA with one repeated factor (time) followed by Sidak’s multiple comparison test.

To determine the major site of action of rolipram, we administered it locally into the left L5 DRG, the left sciatic nerve, the lumbar spinal cord, and the plantar skin of the left hind paw in dose of 0.03 mg. This amount was selected on the basis of the intraperitoneal dose used (3 mg/kg) and of preliminary studies. In a previous report, injection of 3 mg/kg of rolipram increased the mechanical threshold from 0.8 g to 16.3 g, 15.7 g and 11.1 g at 0.5, 1, and 1.5 h after injection, respectively (Kim et al., 2015a). Local administration of rolipram in the L5 DRG significantly increased the mechanical withdrawal threshold compared to the baseline in a dose-dependent manner (**Figures 1A,B**). A 0.03-mg dose of rolipram in the L5 DRG significantly increased the mechanical withdrawal threshold over the baseline level at 0.5, 1, and 1.5 h, with a return to baseline at 2 h after injection (**Figure 1A**). Local administration of 0.03 mg of rolipram into the sciatic nerve or lumbar spinal cord also significantly increased the mechanical withdrawal threshold at 0.5 h (**Figure 1A**). However, local administration of rolipram into the plantar skin nerve terminal had no significant effect on the mechanical threshold (**Figure 1A**). These data indicate that the L5 DRG was a major site of action for rolipram in paclitaxel-induced neuropathic pain in rats.

### Analgesic Effects of db-cAMP in the L5 DRG on Paclitaxel-Induced Neuropathic Pain

Local administration of 0.05 mg of db-cAMP in the left L5 DRG significantly increased the mechanical threshold at 2, 3, 4, and 5 h after injection, with a return to baseline at 6 h (**Figure 1C**). These data indicate that the increase of intracellular cAMP in the DRG produced analgesic effects on paclitaxel-induced neuropathic pain.

### Effects of Paclitaxel and Rolipram on Inflammatory Markers in the DRG

**FIGURE 2:**
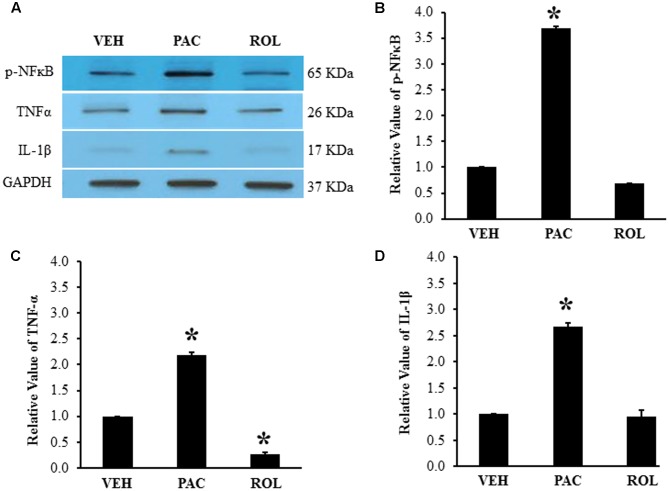
Paclitaxel increased levels of p-NFκB, TNF-α, and IL-1β in rat DRGs. **(A)** Western blot showing the expression of p-NFκB, TNF-α, and IL-1β in DRGs after an injection of vehicle (VEH, *N* = 3) or paclitaxel (PAC, *N* = 3) on day 20 after the first paclitaxel injection. Rolipram (PAC+ROL, 3 mg/kg, *N* = 3) was intraperitoneally injected on day 20 after the first injection of paclitaxel, and the L1–L6 DRGs were obtained 1 h after the injection. **(B–D)** Quantification of p-NFκB, TNF-α, and IL-1β in the DRGs. Paclitaxel increased the levels of p-NFκB, TNF-α, and IL-1β in rat DRGs, and subsequent treatment with rolipram decreased them. The data are expressed as means ± standard deviations for three rats. The asterisks indicate values that are significantly different (*P* < 0.05) from the values for the vehicle group as determined by the Mann–Whitney *U* test.

Paclitaxel administration significantly increased the levels of p-NFκB (3.6 times), TNF-α (2.2 times), and IL-1β (2.7 times) in the lumbar DRGs over levels in the vehicle control groups (**Figures 2A–D**). Subsequent rolipram administration significantly decreased the paclitaxel-increased p-NFκB, TNF-α, and IL-1β levels in the DRGs (**Figures 2A–D**). These results indicate that paclitaxel raised the levels of inflammatory markers in the DRGs and rolipram subsequently decreased them.

### Co-localization of PDE4 and IL-1β in Neurons and Satellite Cells in DRGs

**FIGURE 3:**
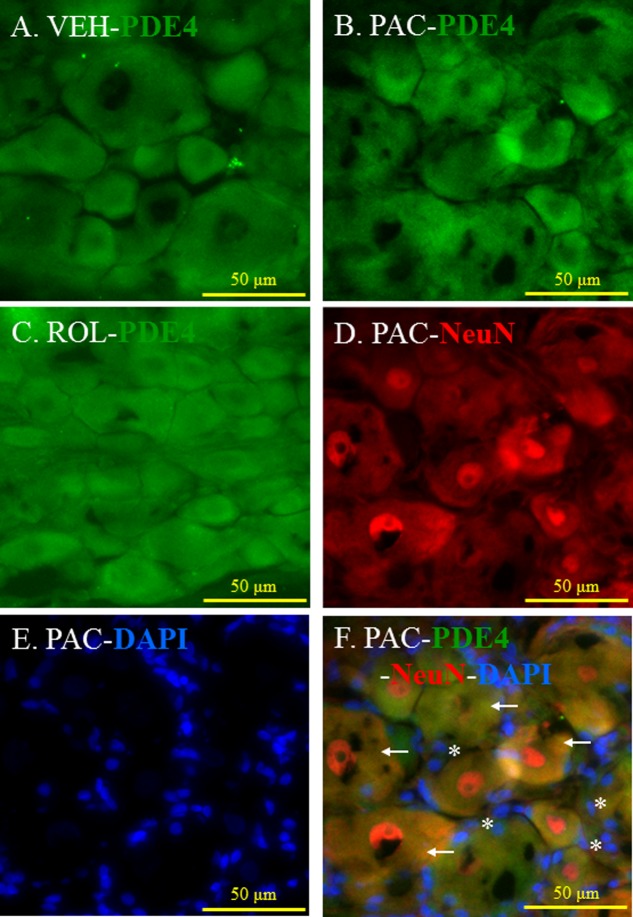
(Representative immunofluorescent images of PDE4) Co-localization of PDE4, NeuN, and DAPI in the L5 DRGs. **(A)** PDE4 (green, Alexa Fluor 488) in the L5 DRG of a vehicle (VEH; 4% dimethyl sulfoxide and 4% Tween 80 in saline)-injected rat. **(B)** PDE4 (green, Alexa Fluor 488) in the L5 DRG of a paclitaxel (PAC; 2 mg/kg/1 ml of vehicle)-injected rat. **(C)** PDE4 (green, Alexa Fluor 488) in the L5 DRG of a paclitaxel + rolipram (ROL; 3 mg/kg)-injected rat. **(D)** NeuN (red, Alexa Fluor 568) in the L5 DRG of a paclitaxel-injected rat. **(E)** DAPI (blue) in the L5 DRG of a paclitaxel-injected rat. **(F)** PDE4 (green, Alexa Fluor 488), NeuN (red, Alexa Fluor 568), and DAPI (blue) in the L5 DRG of a paclitaxel-injected rat. The L5 DRGs of the VEH (*N* = 3) and PAC groups (*N* = 3) were obtained on day 20 after the first paclitaxel injection. For the ROL group, L5 DRGs were obtained 1 h after intraperitoneal injection of rolipram (3 mg/kg) on day 20. PDE4 was expressed in both neurons and satellite cells in the DRG. Stars and arrows indicates satellite cells and neurons, respectively. Scale bars, 50 μm.

PDE4 was expressed in the L5 DRGs of both vehicle- and paclitaxel-treated rats (**Figures 3A,B**). The intensity of PDE4 staining did not markedly differ in the L5 DRG cells of paclitaxel-treated, vehicle-treated, and rolipram-injected paclitaxel-treated rats (**Figures 3A–C**). PDE4 was expressed in small (<30 μm in diameter), medium (30–45 μm), and large (>45 μm) neurons (Scroggs and Fox, 1992). Immunohistochemical staining demonstrated that PDE4 was expressed in small-, medium-, and large-size neurons in the L5 DRG (**Figures 3D,F**). Satellite cells surrounding NeuN-positive neurons were detected with DAPI staining (**Figures 3E,F**). PDE4 was expressed in both NeuN-positive neurons and DAPI-positive satellite cells in the DRG of paclitaxel-treated rats (**Figure 3F**).

### The Intensity of IL-1β in the DRG Tissues

**FIGURE 4:**
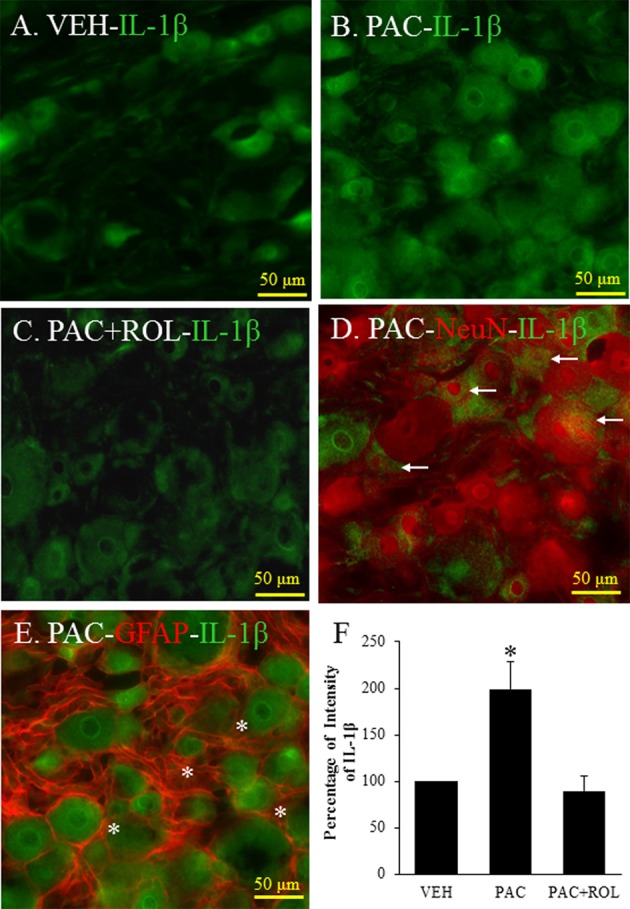
(Representative immunofluorescent images of IL-1 β) Co-localization of IL-1β, NeuN, and GFAP in DRGs. **(A)** IL-1β (green, Alexa Fluor 488) in the L5 DRG of a vehicle (VEH; 4% dimethyl sulfoxide and 4% Tween 80 in saline)-injected rat. **(B)** IL-1β (green, Alexa Fluor 488) in the L5 DRG of a paclitaxel (PAC; 2 mg/kg/1 ml of vehicle)-injected rat. **(C)** IL-1β (green, Alexa Fluor 488) in the L5 DRG of a paclitaxel + rolipram (ROL; 3 mg/kg)-injected rat. **(D)** IL-1β (green, Alexa Fluor 488) and NeuN (red, Alexa Fluor 568) in the L5 DRG of a paclitaxel-injected rat. **(E)** IL-1β (green, Alexa Fluor 488) and GFAP (red, Alexa Fluor 568) in the L5 DRG of a paclitaxel-injected rat. **(F)** Quantification of IL-1β in the DRG. Paclitaxel increased the levels of IL-1β in the DRG, and subsequent treatment with rolipram decreased that. The L5 DRGs of rats in the VEH (*N* = 3) and PAC groups (*N* = 3) were obtained on day 20 after the first paclitaxel injection. For rats in the ROL group, L5 DRGs were obtained 1 h after intraperitoneal injection of rolipram (3 mg/kg on day 20). Rolipram decreased the PAC-increased IL-1β intensity in the DRG. Stars and arrows indicates satellite cells and neurons, respectively. Scale bars, 50 μm. The data are expressed as means ± standard error for three rats. The asterisks indicate values that are significantly different (*P* < 0.05) from the values for the vehicle group or PAC + ROL group as determined by the Mann–Whitney *U* test.

IL-1β was expressed in the L5 DRG in both vehicle- and paclitaxel-treated rats (**Figures 4A,B**). The intensity of IL-1β staining was significantly higher in the L5 DRG cells of paclitaxel-treated rats than in the DRG cells of vehicle-treated rats (**Figures 4A,B,F**). IL-1β was expressed in neurons and satellite cells in the L5 DRG (**Figures 4D,E**). Furthermore, treatment with rolipram significantly decreased the intensity of IL-1β staining in the DRG cells (**Figures 4B,C,F**). These results demonstrate that PDE4 and IL-1β were colocalized in both neurons and satellite cells in DRG cells and rolipram deceased the intensity of IL-1β in the DRG cells.

## Discussion

This study investigated the major sites and mechanisms of rolipram’s analgesic effects in a rat model of paclitaxel-induced neuropathic pain. Local administration of rolipram to the L5 DRG significantly increased the mechanical withdrawal threshold in rats with paclitaxel-induced neuropathic pain for 1.5 h, indicating that the L5 DRG was a major site of action for rolipram in paclitaxel-induced neuropathic pain in rats. Similarly, local administration of db-cAMP to the L5 DRG also significantly increased the mechanical thresholds. Paclitaxel administration significantly increased the levels of the proinflammatory proteins p-NFκB, TNF-α, and IL-1β in the DRG, and rolipram treatment decreased them. These findings indicate that rolipram decreased inflammatory cytokines in the DRGs. We further demonstrated that PDE4 and IL-1β were co-localized in both neurons and satellite cells in the DRG. Taken together, our results suggest that the DRG is a major site of action for rolipram and that rolipram works to reverse chemotherapy-induced neuropathic pain in part by inhibiting inflammatory cytokines.

We previously reported that rolipram had a potent analgesic effect when administered systemically and that systemic rolipram treatment delayed the development of paclitaxel-induced neuropathic pain in rats (Kim et al., 2015a). In the present study, we identified the major site of action of rolipram in chemotherapy-induced neuropathic pain in rats by administering the drug locally at several sites. Local administration of rolipram in the L5 DRG produced the strongest analgesic effects among the sites tested, suggesting that the DRG is a major site of rolipram’s anti-neuropathic pain activity. The DRG contains both pseudounipolar neurons that convey sensory information from the periphery to the spinal cord and satellite cells that surround neuronal bodies (Sapunar et al., 2012). Furthermore, the DRG is located outside of the blood-brain barrier that protects the spinal cord and brain (Sapunar et al., 2012). Therefore, the DRG is vulnerable to the effects of chemotherapeutic drugs such as paclitaxel, cisplatin, vincristine, and bortezomib. These drugs induce neuropathic pain in cancer patients through marked functional impairment of both Aβ and Aδ nerves (Dougherty et al., 2004). In the DRG, chemotherapeutic drugs increase levels of inflammatory cytokines and reactive oxygen species, which contribute to the development and maintenance of chemotherapy-induced neuropathic pain (Cata et al., 2008; Kim et al., 2010, 2016b). Recently, immunoregulatory drugs such as thalidomide and minocycline were shown to decrease paclitaxel-induced neuropathic pain through downregulation of NF-κB and cytokines such as TNF-α (Cata et al., 2008). Reactive oxygen species scavengers such as phenyl-*N*-tert-butylnitrone and 4-hydroxy-TEMPO also decreased paclitaxel-induced neuropathic pain in animals (Kim et al., 2010). Cannabinoid type 2 receptor agonists prevented and reduced allodynia in a rat model of paclitaxel-induced neuropathic pain (Naguib et al., 2008). In addition, our study confirms that selective PDE4 inhibitor reduced chemotherapy-induced neuropathic pain through inhibiting of inflammatory cytokines in the DRGs.

We determined that rolipram exerts its analgesic effects in rats with paclitaxel-induced neuropathic pain by decreasing inflammatory cytokines in the DRG. Rolipram is a selective PDE4 inhibitor. PDE4 degrades the phosphodiester bond in cAMP and then terminates the action of cAMP (Beavo, 1995; Houslay and Adams, 2003). PDE4 is the predominant cAMP-specific PDE in the neurons and glial cells of neural tissues (Iona et al., 1998; Jin et al., 1999) and also predominates in immune cells such as basophils, eosinophils, neutrophils, monocytes, macrophages, and T lymphocytes (Houslay et al., 2005; Zhang et al., 2005). Therefore, rolipram may increase cAMP levels in both nerve and immune tissues, in turn inhibiting NF-κB and decreasing the production of inflammatory cytokines (TNF-α, IL-1β), chemotaxis, and cytotoxicity (Takahashi et al., 2002; Chio et al., 2004). Increases in these inflammatory cytokines can produce pain behaviors (Wagner and Myers, 1996). In our study, both rolipram and db-cAMP, a cAMP analog, showed potent analgesic effects in the rat model of paclitaxel-induced neuropathic pain. Therefore, the inhibition of PDE4 and promotion of cAMP are critical targets for the treatment of chemotherapy-induced neuropathic pain.

We found that PDE4 was localized in the neurons and satellite cells in the DRG. The Aβ fiber, a myelinated large-size neuron, has nociceptors that respond to moderate and noxious pressure or pinch (Perl, 1968). The Aδ fiber is a myelinated medium-size neuron that carries information about mechanical and thermal pain (Roberts and Elardo, 1985). The C fiber, a non-myelinated small-size neuron, includes high-threshold mechanoreceptors with superficial or deep receptive fields (Alloui et al., 2006). We demonstrated that PDE4 was localized in all three of these differently sized neurons, providing further evidence that PDE4 terminated the action of cAMP in the DRG neurons and that rolipram decreased PDE4 activity.

In the present study, paclitaxel treatment increased IL-1β expression in the DRG cells, and rolipram reversed the paclitaxel-induced increase in IL-1β. Paclitaxel has a lipopolysaccharide-like action and accumulates immune cells into the DRG (Manthey et al., 1992; Kim et al., 2016a). The immune cells activated by paclitaxel can produce inflammatory cytokines such as TNF-α and IL-1β through an increase in p-NFκB (Manthey et al., 1992). In addition, these increased amounts of p-NFκB, an activated form of NF-κB, are translocated into the nucleus, where various inflammatory cytokines, including TNF-α and IL-1β, are produced. Rolipram decreased p-NFκB levels in the DRG via inhibition of PDE4 and thereby decreased the release of inflammatory cytokines. Therefore, we conclude that inflammatory cytokines in the DRG are involved in chemotherapy-induced neuropathic pain and that PDE4 may be a critical target for treating this form of pain.

Paclitaxel activates Toll-like receptor 4 (TLR4) through lipopolysaccharide-like action (Manthey et al., 1992). TLR4 is expressed on the cell surface of innate immune cells, small primary afferent neurons and microglia and astrocytes in the central nervous system (Li et al., 2016). Detaily, TLR4 is expressed in CGRP- and IB4-positive small DRG neurons and astrocyte in spinal cord. The activation of TLR4 induces inflammatory cytokines (Li et al., 2014). PDE4 was expressed in the DRG in the present study. Therefore, the interaction of PDE4 and TLR4 in the DRG may be involved in chemothrapy-induced neuropathic pain (Koks et al., 2008).

Some of the limitations of the present study include: (1) This study was performed in an animal model of chemotherapy-induced neuropathic pain. Thus, it will be interesting to see if rolipram has similar analgesic effects on other chemotherapy agents-induced neuropathic pain models. (2) The present study was performed in a small number of rats in each group. Thus, higher numbers of rats are needed to increase the reliability. (3) This study was performed in an animal model. Thus, clinical study be needed.

The present study demonstrated that the local administration of rolipram in the L5 DRG ameliorated marked mechanical hyperalgesia induced by paclitaxel in a rat model of chemotherapy-induced neuropathic pain via inhibition of inflammatory cytokines and PDE4. We thus conclude that the DRG is a site of action of PDE4 inhibitors and that PDE4 inhibitors could be useful in alleviating chemotherapy-induced neuropathic pain in patients with cancer. However, further clinical investigations are needed.

## Author Contributions

HK, S-HH, and SA designed the study, conducted the experiments, analyzed the data, and wrote the manuscript. EO conducted the experiments.

## Conflict of Interest Statement

The authors declare that the research was conducted in the absence of any commercial or financial relationships that could be construed as a potential conflict of interest.
